# Tackling Health Inequity Through Focused Care in a ‘Deep End’ General Practice in England: A Case Study

**DOI:** 10.1177/21501319251370819

**Published:** 2025-11-11

**Authors:** Lily Warburton, Morro Touray, Heather Gage, Nora Mzaoui, Emma Baker, Emma Lopez, Sarah Webb

**Affiliations:** 1 Surrey Health Economics Centre, University of Surrey, Guildford, UK; 2 Wellsbourne Healthcare CIC, Whitehawk Practice, Brighton, UK

**Keywords:** access to care, community health, health inequities, impact evaluation, primary care, prevention, social determinants of health

## Abstract

The National Health Service (NHS) in England provides funding for social prescribing to address the social determinants of ill-health. This paper describes a case study of how one general practice serving a disadvantaged population transitioned to Focused Care, a more intensive form of social prescribing, to increase engagement and improve outcomes. Characteristics of service users and their presenting problems, rates of engagement, resource implications of the service and potential benefits are discussed.

## Background

First popularised in the United Kingdom (UK), social prescribing is now adopted in many countries worldwide as a means by which community-based health professionals, including family medicine doctors or general practitioners, can address the individual social needs of their patients.^1,2^ Often called ‘community referral’ or ‘linkage schemes’, social prescribing enables health professionals to refer patients to advisers (variously called link workers, navigators or care coordinators) for an assessment of their needs and agreement of a co-produced goal-led care plan that either involves signposting or introducing clients to suitable agencies in the community (the ‘delivery arm’) with potential to provide appropriate advice and support.^3-5^ Social prescribing aligns with the bio-psycho-social model of health; its theoretical principles are grounded in self-identity, resilience, motivation and coping with complex issues.^6^

Social prescribing seeks to address ‘the causes of the causes of ill-health’ as emphasised by the Marmot report in 2012 which brought empirical clarity to the relationship between inequalities in wider social, economic and environmental factors and inequalities in health outcomes in England.^7^ Over the ensuing few years, a heterogeneous array of social prescribing schemes were introduced which local evaluative studies (albeit often weak methodologically) generally reported to have favourable effects on wellbeing.^8^ The concept gathered significant momentum and interest amongst policy makers such that the Long-Term Plan for the National Health Service in 2019 provided for social prescribing link workers to be introduced universally across England through dedicated central funding for Primary Care Networks (groupings of general practices providing primary medical services to between 30 000 and 50 000 people).^9^

This paper describes the evolution of social prescribing in a large general practice serving a disadvantaged population in the south of England. The practice included localities that are in the top 2% most deprived in England. People living in this area struggle with issues such as substandard housing, food insecurity, unemployment and poor literacy. Over 60% of patients registered with the practice live with at least one long-term condition. The prevalence of COPD, learning disabilities and mental health conditions is more than double the national average.

## Social Prescribing and Focused Care in a ‘Deep End’ practice

Prior to the national initiative, the study practice offered a social prescribing service that was funded through local commissioning. A Social Prescribing Link Worker and a Health Engagement Worker collaboratively ran the service alongside the regular clinical and administrative staff that included a mental health worker, a community pharmacist and a community projects manager. Social prescribing was a whole team effort and normalised in the daily work of the practice^10^; reception staff were trained in active signposting (e.g. to local food banks) whilst doctors, nurses, therapists and healthcare assistants could make direct social prescriptions to community support activities (e.g. exercise classes) when delivering care for chronic conditions.

An internal audit of the 183 clients receiving a social prescription between March and October 2020 revealed that, amongst many positive outcomes, around one third of patients did not follow up on the original referral to the service. This finding prompted the launch of a consultation exercise involving patients and staff that ended with a plan for service developments. Prominent amongst the changes was the decision to adopt a Focused Care approach, as designed by Hope Citadel Healthcare, a not-for-profit group running NHS-funded general practices in north-west England. Focused Care concentrates on whole-person care, early disease detection and equipping clinicians to address social determinants of health. Emerging evidence from other ‘Deep End’ practices (i.e. in areas of social deprivation) was showing how Focused Care was successful in increasing engagement of previously ‘invisible’ patients and improving outcomes. Through a partnership with the Focused Care Community Interest Company,^11^ the Link Worker and Health Engagement Worker were trained and mentored to deliver enhanced provision rooted in the principles of social prescribing. They were renamed as Focused Care Practitioners with a remit to concentrate on the most complex cases; patients with less complex needs were referred to the core social prescribing service provided by the local Primary Care Network.

Focused Care seeks to directly address the Inverse Care Law in which healthcare provision favours more articulate and assertive individuals and thereby compounds the disadvantage of the people with poorest health.^12^ Through a holistic and flexible approach, often working with whole households to unpack situations, Focused Care can offer extended consultations and a responsive, needs-based commitment to clients, delivered within clearly defined professional boundaries and under appropriate clinical supervision. It can therefore provide a level and intensity of input above that of the usual social prescribing offer that is, though flexible, usually 6-12 contacts over a 3-6 month period and limited to linking individuals with other agencies to provide the support.^4^ Like regular social prescribing, Focused Care Practitioners leverage local health and community resources around clients but through ongoing monitoring and follow-up seek to ensure that appointments are attended, and that practical and emotional support reaches families.^13^

This paper reports an analysis of the first 37 months of operation of Focused Care at the study practice (January 2021 to January 2024). The aim was to review the profile of clients and activity undertaken. Given the potentially high levels of input afforded to individual clients, the resource implications of the service were also explored to inform future policy and practice. Whilst limited data on outcomes were available, the analysis centred on the characteristics of clients, engagement, reasons for referral, the issues requiring the most input and the costs and potential benefits of the service.

## Methods

The study was a single site case study design using mixed methods.^14^ Records from the Focused Care service at the study site were accessed retrospectively by members of the practice, anonymised by assigning a unique study identifier to each client, cleaned and organised in Microsoft Excel and then transferred to the research team for analysis. The dataset contained quantitative and qualitative variables. Where required, quantitative variables such as age were separated into discreet groups, to allow for better visualisation of characteristics and aid analysis. Ethnicity was simplified into the five high-level groups as used in the census. Categorical variables such as gender were coded; main reason for referral was based on groupings provided by the practice.

Coded variables (client age, gender, ethnicity, first language, main reason for referral, number of contacts, time in the system) were analysed statistically in SPSS v29.0.1.0 to produce descriptive tables and crosstabulations. A more detailed analysis of open text reporting of practitioner communications with clients and actions taken was conducted for a sample of clients that were selected using the random number generation tool within the clinical system. Interactions with individual clients were reviewed and exemplars were chosen to illustrate variety in client needs and interventions. Resource implications of the findings were considered and discussed.

The work involved analysis of anonymised records, and the NHS Health Research Authority decision tool indicated that ethical approval was not required. Best practice processes to ensure research integrity were applied throughout.

## Findings

There were 734 patients who received referral to Focused Care in the 37 month period; of these, 609 were completed cases (125 were still open/ongoing at the data collection end point). Data are summarised in Table 1.

**Table 1.** Characteristics of Patients, Number of Contacts and Time Case Was Active.

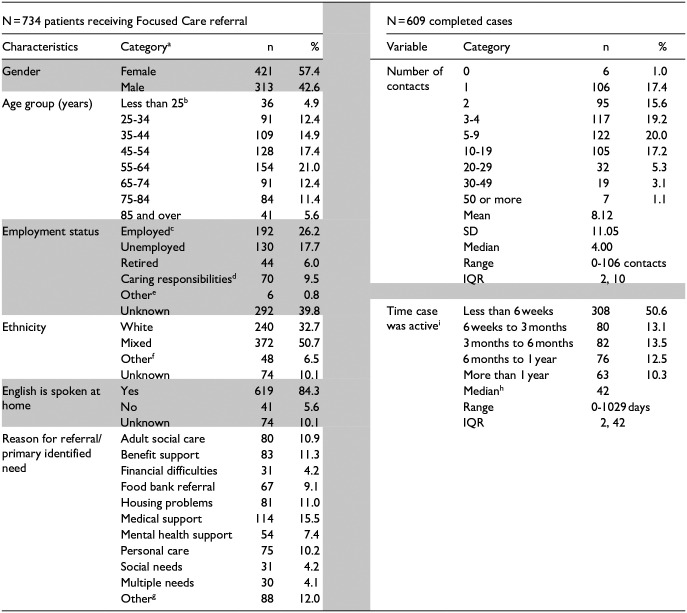							

a Categories are amalgamations of finer groupings.

b n = 10 people less than 18 years of age assessed in a family setting.

c Of those employed, n = 63 were health professionals.

d Amongst carers, n = 8 were home makers with children.

e n = 2 were students, n = 4 were on long term sick leave.

f n = 20 were Black, 10 were Asian.

g Other includes child safety, crime victim, domestic abuse, safeguarding check, employment support, not specified and no contact.

h Non normal distribution so mean not valid.

i Pearson correlation: number of contacts and time case was active, *r* = .44, *P* < .001.

The shading indicates breaks between variables.

*Client characteristics*: Amongst the full sample of 734, referrals of women were more frequent than of men. The smallest proportions of clients were in the youngest age group, otherwise the distribution across ages was relatively even; half of clients were described as having mixed ethnicity. Main language was unknown for 10% of referrals but where this was recorded, English was not the first language for 5% of clients. In addition, poor literacy skills were reported for some native English speakers. Employment status was poorly recorded but, where this was given, about one quarter of clients were in employment. There was a range of primary reasons recorded for referral with none standing out as more important from the rest. People referred for adult social care were mostly over 60 years whereas other reasons were more evenly spread across the age groups. In general, the characteristics of clients matched the national profile of social prescribing recipients.^15,16^

*Engagement and contacts*: Engagement with the service was high with practitioners unable to contact only 1% of people referred. Considering the 609 completed cases (defined as when the relevant support system is in place and the client is discharged), around three quarters involved fewer than 10 contacts (face-to-face meetings, phone calls, video meetings or texts); one half were resolved within 6 weeks and three quarters within 6 months. Time in the system was positively and significantly correlated with number of contacts. The most resource intensive decile of clients had 20 or more contacts; based on time receiving care, the top decile were all still in the system after 12 months. Having more contacts was significantly associated with being referred for multiple needs; age, gender, ethnicity, employment status, first language were not related to number of contacts (data not shown). For many clients the main presenting reason was the ‘tip of the iceberg’ and other problems were uncovered when practitioners undertook initial assessments.

*Client examples*: The more detailed analysis covered the activity associated with 43 randomly selected clients. This sample was slightly younger on average that the full 734 participants (51 vs 55 years) and had slightly more contacts (average of 8 vs 5). Six completed cases, selected for variability in complexity and duration of care, are described in Table 2.

**Table 2.** Case Studies of Focused Care.

Case	Number of contacts; time in system	Client characteristics	Reasons for referral	Actions and outcomes
1	1 contact 1 day 240 min	80 years White British Retired male	Medical needs	Pharmacy reported medications not collected. District Nurses couldn’t make contact. Police called to gain access finding patient on the floor. Paramedics called.
2	2 contacts 4 weeks 60 min	49 years British male Mixed ethnicity	Financial difficulties due to unemployment	Efforts to get work through usual channels had failed – client felt due to discrimination. Referred to local employment project for non-white ethnic groups where he secured employment
3	42 contacts 6 months 880 min	40 years White female (European) Employed	Multiple – Home Office debt; cannot find dentist; domestic violence/ safeguarding	Supported to contact Home Office and find dentist. Social worker assigned to family. Help to client to find alternative work away from partner
4	33 contacts 11 months 1000 min	33 years African female English not first language Occupation not stated	Initial referral for financial difficulties, but also ongoing court case	Referral to money advice centre, foodbank and for housing support. Local advocacy service arranged to attend police interviews and court and translate legal documents
5	58 contacts 14 months 1160 min	70 years British male Mixed ethnicity Retired	Social Need/isolation is initial reason for referral but assessment reveals fragility and falls, unsafe home environment, financial difficulties	Referral to befriending service. Occupational therapy assessment and ultimate move to supported living and new care package organised. Referral to foodbank and Citizens Advice Bureau. Help with application for Personal Independence Payment. Supported access to primary and secondary health care appointments.
6	23 contacts 18 months 120 min	59 years White male British Unemployed	Financial difficulties, leading to anxiety	Counselling and provision of information. Client takes up carpentry course and attends art group, photography walks and local gym. Client also starts to Provide gardening and handyman type services in local community.

Cases 1 and 2 are examples of where help could be provided relatively quickly and with low levels of input for clearly defined issues. Case 1 also indicates the value of the proactive team approach enabled by whole practice ownership of Focused Care and full integration of the Focused Care Practitioners in the practice team. Case 2 indicates the importance and value of practitioners as expert sources of local knowledge and contacts.

The other four cases involved support for clients over many months because of complexity in their lives. These cases indicate the breadth of problems encountered (finance, justice, physical and mental health, social issues, safeguarding) and underscore the importance of working with local organisations in both the statutory and voluntary sectors to deliver the assistance and support that is required. Whilst some cases involved intensive input (e.g. Case 3 which had 42 contacts over 6 months), Focused Care also provides low level support for vulnerable individuals over a relatively long period of time (e.g. Case 6).

*Resource implications*: The Focused Care service was delivered with 1.75 FTE Agenda for Change Band 5 level staff, equivalent to a high level clinical psychology assistant practitioner or an entry level counsellor, at a total annual cost (in British pounds, 2024) to the practice of some £115,000.^17^ Based on the total of 734 clients over the 37 month period, the average cost per client is approximately £470, although this hides a lot of variability. Moreover, this cost to the practice does not take account of the indirect costs associated with the supervision of the practitioners and the input of other professionals and agencies in both the formal and voluntary sectors to which clients were referred. However, Focused Care also generates offsetting benefits for the practice and wider health system: clinical time is saved because Practitioners manage social complexity; enhanced engagement boosts practice achievement on the Quality and Outcomes Framework (a associated performance-related payments mechanism); early intervention reduces A&E visits, unplanned hospital admissions and non attendance of outpatient appointments.

## Discussion

The Focused Care approach enables practitioners to provide open ended support to clients, many of whom have multiple complex problems. Focused Care Practitioners were successful in proactively contacting nearly all patients referred to the service thereby significantly improving on the engagement rate of the earlier social prescribing scheme in which about one third of patients were lost to follow up. The Focused Care Practitioners offered tailored support in the form of signposting or direct referrals to appropriate services and tangible assistance, for example, with completing applications for benefits or accompanying clients to medical or legal appointments. They continued to work with clients until the problems identified were addressed; they could reopen cases as and when needed.

Focused Care offers the ultimate personalised service but has the potential to be resource intensive for practices at an individual level, particularly when supporting clients with multiple needs requiring intensive input. Additional costs arise when local services and organisations (statutory and voluntary) are mobilised to assist or support clients. High levels of need in areas of social deprivation raises issues around resourcing of Focused Care services and the capacity of local organisations to support successive people referred. Making a business case for funding can be challenging because many benefits from holistic care, such as improved outcomes, savings in clinical time, strengthened team working, better cross-sectoral working and mobilisation of external statutory and voluntary services, are difficult to quantify.

A strength of the study is that it highlights Focused Care as a new and extended version of social prescribing in the pursuit of greater health equity. It also identified the need for further research. The work is limited in several respects. It relied on retrospective records that were not designed for systematic measurement and reporting of outcomes; some data on client characteristics were missing. Future studies taking a prospective approach should include assessment of individual level impacts on knowledge, skills, motivation, resilience and control, in addition to improved health and wellbeing and the client experience. It is well recognised, however, that efforts to evaluate social interventions face significant challenges due to multisectoral involvement, the heterogeneity of client needs and outcomes. and the longitudinal (often intergenerational) and intangible nature of many of the effects.^3,18,19^ In addition, societal benefits arise from improved equity and social cohesion, reduced expenditures on health and other social services and from productivity gains when clients are helped back into employment.^1,20^ Various attempts to measure such effects report markedly different social rates of return on investment depending on the programmes evaluated, the scope of the investigation, the range of benefits included, and valuations adopted.^19^

A report in the UK in 2015^21^ identified that 20% of consultations with general practitioners were about social problems. Hence social prescribing was viewed as a means of reducing demand on pressured primary care health services. Use of link workers to assess people presenting with non-medical issues is less costly than general practitioner consultations. Moreover, link workers signpost or refer clients out of primary care to a range of community-based services for ongoing support.^22,23^ Further limitations of the work reported in this paper are that it did not explore changes in utilisation of general practitioner services or take the whole system perspective that would capture cost shifting between agencies.^24,25^ Several confounding factors would have hindered interpretation of trends in general practitioner consultations, including service delivery changes following the pandemic and the fact that some patients were referred to Focused Care because of poor engagement with health services, rather than overuse.

Tracking the whole system costs and effects associated with onward referrals is complex and a large and well-resourced study is required to do this comprehensively. Future studies should also include a comparison of the characteristics and outcomes of the clients referred for Focused Care with those receiving the regular social prescribing service to improve understanding of the value added from Focused Care and inform stratification. There is also a need to explore the lower referral rate amongst the youngest age band observed in this and other studies, and specifically whether it reflects a lower prevalence of social issues in this group or limitations in identifying and referring individuals which needs to be addressed by training.

## Conclusions

Social prescribing is viewed as a way of reducing social inequities in health by addressing the social determinants,^20^ but concerns have been raised that individuals from disadvantaged backgrounds are less likely to access services and have a reduced ability to engage and benefit from them.^26^ It is in this respect that the more intensive, personalised and holistic approach of Focused Care expects to be effective with hard-to-reach individuals.

Ultimately the ability of services to provide for clients depends on the resources available and some prioritisation may be necessary in areas of high need. The open-ended nature of interventions offered by Focused Care has potential to be resource intensive at an individual level and it is only possible to speculate about the future costs saved for the health and social care system and other benefits of improved wellbeing enjoyed by the service recipient. Such imponderables mean decision making about the allocation of resources is problematic, including judging whether the additional input from Focused Care is good value-for-money compared to a lighter touch.^27^ Research to explore these challenges requires a robust conceptual framework^28^ that goes beyond economic factors to incorporate society’s moral, ethical and philosophical values.
